# Accuracy of high-frequency oscillations recorded intraoperatively for classification of epileptogenic regions

**DOI:** 10.1038/s41598-021-00894-3

**Published:** 2021-11-01

**Authors:** Shennan A. Weiss, Richard J. Staba, Ashwini Sharan, Chengyuan Wu, Daniel Rubinstein, Sandhitsu Das, Zachary Waldman, Iren Orosz, Gregory Worrell, Jerome Engel, Michael R. Sperling

**Affiliations:** 1 Department of Neurology, State University of New York Downstate, Brooklyn, NY 11203 USA; 2 Department of Physiology and Pharmacology, State University of New York Downstate, Brooklyn, NY 11203 USA; 3 Department of Neurology, New York City Health + Hospitals/Kings County, Brooklyn, NY USA; 4 Department of Neurology, Mayo Systems Electrophysiology Laboratory (MSEL), Rochester, USA; 5 Department of Physiology and Biomedical Engineering, Mayo Clinic, Rochester, MN 55905 USA; 6 Department of Neurology, David Geffen School of Medicine at UCLA, Los Angeles, CA 90095 USA; 7 Department of Neurobiology, David Geffen School of Medicine at UCLA, Los Angeles, CA 90095 USA; 8 Department of Psychiatry and Biobehavioral Sciences, David Geffen School of Medicine at UCLA, Los Angeles, CA 90095 USA; 9 Brain Research Institute, David Geffen School of Medicine at UCLA, Los Angeles, CA 90095 USA; 10 Department of Neurology and Neuroscience, Thomas Jefferson University, 901 Walnut St. Suite 400, Philadelphia, PA 19107 USA; 11 Department of Neurosurgery, Thomas Jefferson University, Philadelphia, PA 19107 USA; 12 Penn Image Computing & Science Lab, University of Pennsylvania, Philadelphia, PA 19143 USA

**Keywords:** Epilepsy, Diagnostic markers

## Abstract

To see whether acute intraoperative recordings using stereo EEG (SEEG) electrodes can replace prolonged interictal intracranial EEG (iEEG) recording, making the process more efficient and safer, 10 min of iEEG were recorded following electrode implantation in 16 anesthetized patients, and 1–2 days later during non-rapid eye movement (REM) sleep. Ripples on oscillations (RonO, 80–250 Hz), ripples on spikes (RonS), sharp-spikes, fast RonO (fRonO, 250–600 Hz), and fast RonS (fRonS) were semi-automatically detected. HFO power and frequency were compared between the conditions using a generalized linear mixed-effects model. HFO rates were compared using a two-way repeated measures ANOVA with anesthesia type and SOZ as factors. A receiver-operating characteristic (ROC) curve analysis quantified seizure onset zone (SOZ) classification accuracy, and the scalar product was used to assess spatial reliability. Resection of contacts with the highest rate of events was compared with outcome. During sleep, all HFOs, except fRonO, were larger in amplitude compared to intraoperatively (*p* < 0.01). HFO frequency was also affected (*p* < 0.01). Anesthesia selection affected HFO and sharp-spike rates. In both conditions combined, sharp-spikes and all HFO subtypes were increased in the SOZ (*p* < 0.01). However, the increases were larger during the sleep recordings (*p* < 0.05). The area under the ROC curves for SOZ classification were significantly smaller for intraoperative sharp-spikes, fRonO, and fRonS rates (*p* < 0.05). HFOs and spikes were only significantly spatially reliable for a subset of the patients (*p* < 0.05). A failure to resect fRonO areas in the sleep recordings trended the most sensitive and accurate for predicting failure. In summary, HFO morphology is altered by anesthesia. Intraoperative SEEG recordings exhibit increased rates of HFOs in the SOZ, but their spatial distribution can differ from sleep recordings. Recording these biomarkers during non-REM sleep offers a more accurate delineation of the SOZ and possibly the epileptogenic zone.

## Introduction

For patients with medically refractory epilepsy who are candidates for epilepsy surgery, invasive intracranial EEG (iEEG) monitoring is often required. Intraoperative electrocorticography (ECoG) using subdural electrodes^1^, and occasionally intrahippocampal depth electrodes^2,3^, are often used in place of prolonged iEEG recordings in childrenn^4,5^, and in adults with a clear pathological substrate^1,6,7^. It is unclear if acute recordings from stereo EEG (SEEG) electrodes could similarly be used to reduce or obviate the need for prolonged epilepsy monitoring unit (EMU) evaluations to capture seizures.

ECoG can identify brain regions with high rates of inter-ictal epileptiform spikes^1^ and help localize the epileptogenic zone (EZ), which is the hypothetical region necessary and sufficient for seizure generation^8^. Results from several studies suggest high-frequency oscillations (HFOs: ripples 80–250 Hz, fast ripples 250–600 Hz)^3,4,6,7,9–11^ is a more specific biomarker of EZ than interictal spikes. Ripples occur more frequently than fast ripples but are thought to be less specific, because they are more often generated by physiological mechanisms^9,10^. Several groups have reported that unresected fast ripples in intraoperative ECoG recordings predict poor post-operative seizure outcome^3,4,6,7,11–15^. When spikes and HFOs coincide, or ripples and fast ripples coincide^15^, the biomarker is thought to be even more specific for EZ^3,16–18^.

Few studies have compared the spatial accuracy of spikes and HFOs in intraoperative ECoG recordings to extraoperative prolonged recordings for identifying the EZ^19–23^. Studies have found anesthesia and analgesia can affect the rate of these biomarkers^24^, and also the spatial distribution with respect to the EZ^23^. A recent meta-analysis^22^ suggests that these studies report conflicting results. Whether anesthesia affects the morphology of HFOs is also unknown. Since electrocorticography is commonly performed, and HFOs may replace or supplement spikes in tailoring resection during ECoG^25^, the accuracy of spikes and HFOs for the EZ in intraoperative recordings must be further clarified with respects to the influence of anesthesia and analgesia.

Prolonged recordings using subdural or SEEG electrodes are advantageous compared to intraoperative recordings, because they are much more likely to capture spontaneous seizures that specify the seizure onset zone (SOZ). The EZ may not always encompass all of the SOZ due to both volume conduction and a possible ictal core region^26,27^, and conversely localizing the SOZ may not identify the complete EZ because of limited temporal sampling during the EMU evaluation. Evidence suggests that HFOs recorded from prolonged implants during non-rapid eye movement (non-REM) sleep may identify the EZ better than the SOZ^28,29^. One contentious issue is whether the spatial distribution of HFO rates during non-REM sleep is stable^15,30,31^. One recent large study found that the classification accuracy of fast ripples for the EZ was higher when a full night of non-REM sleep was analyzed as opposed to 5-min segments^32^. This issue is of particular importance to comparing intraoperative to sleep recordings, since the duration of the intraoperative recordings are intrinsically limited.

In this study, we used intraoperative and extra-operative non-REM sleep recordings from SEEG electrodes, to determine whether the morphology of HFOs is altered by anesthesia. We also compared the rates of HFOs and sharp-spikes (i.e. spikes that when digitally filtered have HFO frequency content 80–600 Hz, but do not have a distinct HFO on time frequency analysis)^3,33,34^, recorded in the two conditions, with each other and the location of both the clinically defined SOZ and the EZ. The EZ was defined as those electrode contacts with the highest rates of events, and then assessing whether removal of these electrodes or EZ was predictive of the post-operative seizure outcome. Our goal was to determine whether intraoperatively recorded HFOs using SEEG electrodes could obviate the need for prolonged iEEG.

## Results

### Patient characteristics, anesthesia, and analgesia

A total of 16 patients with medically refractory epilepsy were included in this study, 11 of them were male (Table S1). The patients had diverse etiologies of their epilepsy (Table S1) with 25% who had non-lesional epilepsy on MRI. Four patients had mesial-temporal lobe epilepsy (MTLE); 2 patients had lateral temporal lobe epilepsy; 4 patients had temporal lobe epilepsy plus other region(s); and 6 patients had extra-temporal lobe epilepsy (Table S1). In the operating room, 2 of the patients were anesthetized with ~ 1% exhaled sevoflurane, 12 patients with ~ 2% exhaled sevoflurane, and 2 patients with propofol 90 mcg/kg/min. As analgesia 7 patients received a continuous infusion of ~ 0.8 ug/kg/h remifentanil, 3 patients received ~ 1.6 ug/kg/h remifentanil, 1 patient received 0.05 ug/kg/h sufentanil. Five patients did not receive analgesia after induction (Table S1).

### Differences in HFO power and frequency during intraoperative recordings compared to non-REM sleep

**Figure 1:**
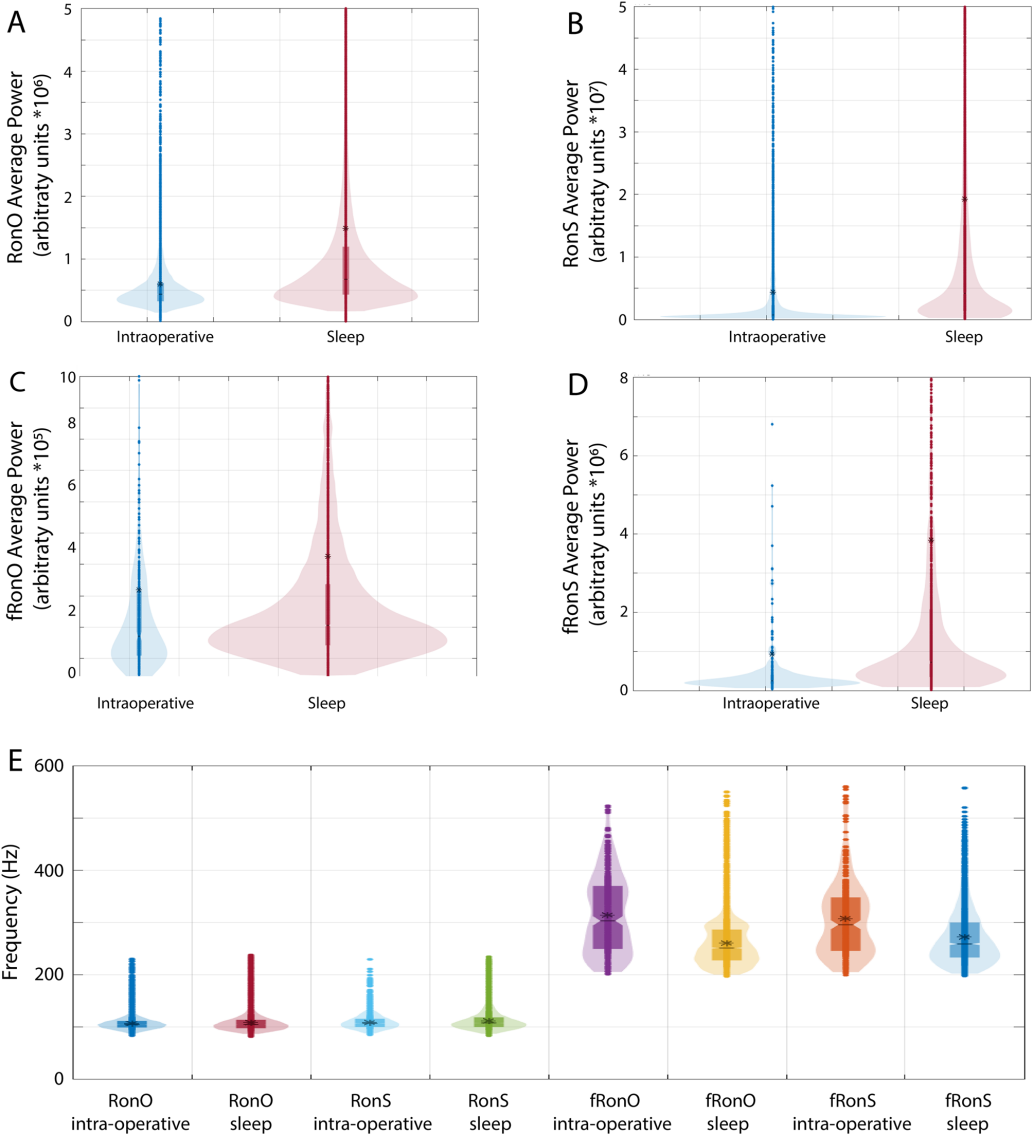
Differences in HFO average power and frequency by recording condition (intraoperative vs. non-REM sleep). Violin plots of ripples on oscillations (RonO, **A**), ripples on spikes (RonS, **B**), fast ripples on oscillations (fRonO, **C**), and fast ripples on spikes (fRonS, **D**) average power. HFO power was greater for all the event types, except fRonO, during non-REM sleep (red) than intraoperative recording (blue, *p* < 0.01). (**E**) Violin plots of HFO spectral frequency. There was a small, but significant, increase in RonO and RonS frequency (*p* < 0.01) and decrease in fRonO and fRonS frequency (*p* < 0.01) during non-REM sleep than during intraoperative recording. Asterisk indicates mean.

Since we quantified the power and spectral frequency of each HFO event, we first asked if anesthesia effected these measures. We used a generalized linear mixed-effects model to account for inter-contact differences in the matched electrodes (n = 530). We found that HFO power was decreased in the intraoperative recordings relative to the non-REM recordings for all HFO types, except for fRonO (*p* < 0.01, Fig. 1, Table 1). We next asked if this difference was influenced by anesthesia type. For RonO, we found the largest relative differences in the mean power for sevoflurane 2% and propofol compared to sevoflurane 1% (Figure S1, Table S2, *p* < 0.05). However, this comparison was indirect such that anesthesia related differences in ripple power were not compared across the intraoperative condition. Furthermore, the two patients that received sevoflurane 1% had smaller RonO power during sleep than the others, which could be related to their poor outcome (Table S1).

**Table 1:** Results of generalized linear mixed-effects models fitting HFO frequency and power.

Response variable	Intercept estimate	Intercept *p* value	Condition estimate	Condition *p* value	SOZ estimate	SOZ *p* value	d.f
RonO Power	12.287 [12.2–12.4]	< 1e−4	1.291 [1.14–1.3]	< 1e−4	0.263 [0.08–0.4]	< 0.005	196,470
RonO Freq	4.734 [4.71–4.75]	< 1e−4	0.03 [0.027–0.032]	< 1e−4	− 0.025 [− 0.05 to 0]	0.08	196,470
RonS Power	14.912 [14.6–15.2]	< 1e−4	0.97 [0.77–1.17]	< 1e−4	− 0.101 [− 0.46 to .26]	0.58	31,479
RonS Freq	4.678 [4.66–4.68]	< 1e−4	0.022 [0.016–0.029]	< 1e−4	0.003 [− 0.013 to .02]	0.7	31,479
fRonO Power	12.251 [11.7–13.3]	< 1e−4	0.3 [− 0.5 to 1.11]	0.47	0.303 [0.03–0.57]	0.03	8416
fRonO Freq	5.755 [5.74–5.77]	< 1e−4	− 0.1877 [− 0.2 to − 0.17]	< 1e−4	− 0.001 [− 0.02 to − 0.004]	0.003	8416
fRonS Power	13.602 [12.6–14.5]	< 1e−4	1.208 [0.4–2.02]	0.004	− 0.209 [− 1.0 to .63]	0.62	2803
fRonS Freq	5.706 [5.68–5.72]	< 1e−4	− 0.115 [− 0.13 to − 0.09]	< 1e−4	0.029 [0.013–0.045]	< 1e−4	2803

The random-effect term was electrode, the fixed effects were recording condition (intraoperative vs. non-REM sleep), and SOZ. Brackets indicate 95% confidence interval.

RonO and RonS spectral frequency was slightly decreased in the intraoperative recordings relative to the sleep recordings, and fRonO and fRonS frequency was increased in the intraoperative recordings (*p* < 0.01, Fig. 1, Table 1). We also examined whether the SOZ influenced HFO power and frequency, and found a relatively weak but sometimes significant correlation (Table 1).

### Effects of non-REM sleep and anesthesia dose and type on HFO rate in SOZ and non-SOZ

**Figure 2:**
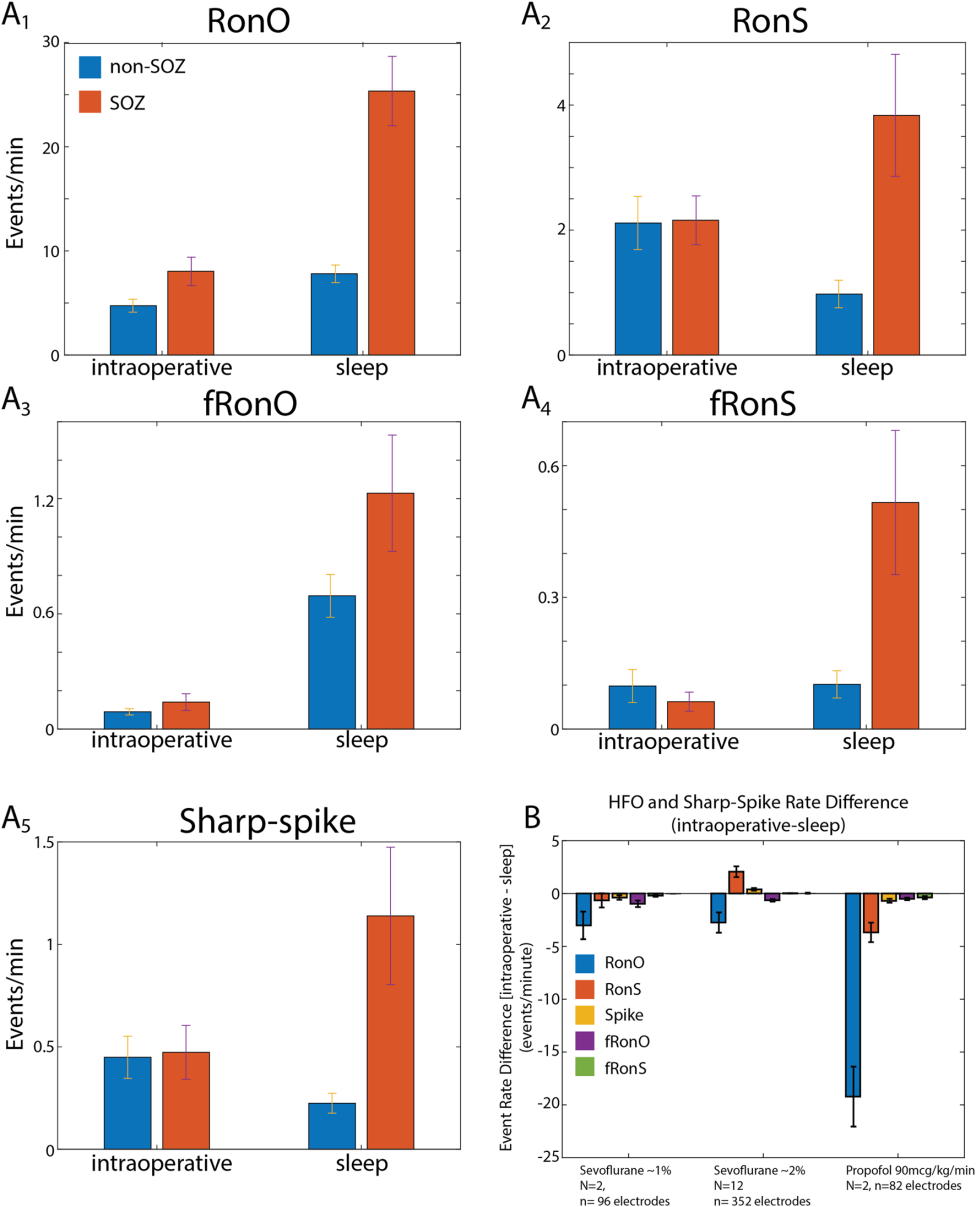
Mean HFO and spike rates in relation to the SOZ, recording condition, and anesthesia. (**A1–5**) A comparison of mean event rates in the non-SOZ (blue) and SOZ (orange) in the intraoperative (left) and sleep (right) condition measured from matched electrode contacts. The rate of RonO (A1), fRonO (**A3**), and fRonS (**A4**) was higher during sleep than intraoperative recording (rmANOVA, *p* < 0.05, N = 16 patients, d.f. = 523,1,1,2 contacts). For both conditions combined, the rate was higher in the SOZ than the non-SOZ for all the individual biomarkers (*p* < 0.01). However, the difference in the rate of HFOs between SOZ and NSOZ was larger during sleep than intraoperative recordings (**A1–5**, *p* < 0.05). (**B**) Differences in HFO and spike rates (intraoperative minus sleep) for each matched electrode contact with respect to anesthesia type and dose. In most cases, rates of HFO were lower during anesthesia than sleep, indicated by negative values in bar chart. Note RonS and spikes were higher during 2% sevoflurane than non-REM sleep. Error bars indicate standard error of the mean (S.E.M).

We next asked for each of the HFO subtypes and sharp-spikes: (1) whether rates differed in the intraoperative and non-REM sleep condition; (2) whether rates were increased in the SOZ; (3) if the intraoperative and non-REM sleep recordings differed in the magnitude or significance of this effect; and (4) whether anesthesia and analgesia selection were significant factors interacting with this effect. We found that rates of some of the HFO subtypes were comparatively increased during the non-REM sleep recordings compared to the intraoperative recordings in the matched contacts (repeated measures two-way ANOVA RonO: F = 113.9, *p* < 0.001; fRonO: F = 36.15, *p* < 0.001; fRonS: F = 26.34, *p* < 0.001, Fig. 2A, Figure S2 d.f. = 524,1,2,1,2). However, the rate of RonS (F = 6.79, *p* < 0.01) and sharp-spikes (F = 12.68, *p* < 0.001) was higher in the intraoperative recording than during the sleep recording. The rate of HFOs and sharp-spikes was higher in the SOZ than in the non-SOZ during the combined sleep and intraoperative recordings (RonO: F = 41.16, *p* < 0.001; RonS: F = 9.97, *p* < 0.005; sharp-spike: F = 13.81, *p* < 0.001; fRonO: F = 7.0124, *p* < 0.01; fRonS: F = 12.10, *p* < 0.001, Fig. 2A). The difference in rates was larger for all event during non-REM sleep than intraoperative recording (RonO:F = 35.65, *p* < 0.001; RonS: F = 4.52, *p* < 0.05; sharp-spike: F = 9.66, *p* < 0.005; fRonO: F = 5.44, *p* < 0.05; fRonS: F = 14.05, *p* < 0.001, Fig. 2A).

HFO generation has been shown to vary by neuroanatomic region^35–39^. We investigated if HFOs and sharp-spikes rates varied by neuroanatomical lobe, and if this location interacted with the effect of the recording condition and the SOZ in the matched contacts. We found that the neuroanatomic location, by lobe, significantly influenced HFO and sharp-spike rates (Figure S3, Table S3). In contrast to other regions, higher ripple and sharp-spike rates, were seen in the frontal lobe, and to a less extent the parietal lobe, in the intraoperative recording relative to the sleep recording (Figure S3). Consequently, for all biomarker rates, except fRonO, the neuroanatomic location of the contact significantly interacted with the recording condition (Table S3). A weak but significant (*p* < 0.05) interaction was found between location and the SOZ for ripples and sharp-spikes only (Table S3). For all the biomarkers, the three-way interaction between SOZ, location, and condition was not significant. General anesthesia dose and type interacted with recording condition (intraoperative vs. non-REM sleep) to influence HFO rates for all HFO subtypes (RonO:F = 15.35, *p* < 0.001; RonS: F = 8.43, *p* < 0.001 sharp-spike: F = 8.24, *p* < 0.001; fRonO: F = 4.52, *p* < 0.05; fRonS: F = 5.45, *p* < 0.01, Fig. 2B). For sharp-spikes, fRonO, and fRonS, but not RonO or RonS, there was also a three-way interaction (*p* < 0.05) between SOZ, anesthesia type, and the recording condition. However, we did not investigate this interaction further because of the small number of patients in this study.

The greatest decrease in HFO and spike rates was seen with propofol anesthesia, whereas higher concentrations of sevoflurane increased the rates of RonS and sharp-spikes (Fig. 2B). Visual analysis of the annotated iEEG revealed that ~ 2% sevoflurane anesthesia often resulted in high amplitude sharply contoured rhythmic oscillations that were sometimes recognized as spikes by the detector (Figure S4), whereas propofol at a dose of 90 mcg/kg/min did not produce a burst suppression pattern.

Analgesia infusion selection also significantly interacted with the condition of the recording for all HFO types and spikes, except RonO (*p* < 0.05). Paradoxically RonS and sharp-spike relative rates were higher in the patients who received no analgesia after induction, Figure S5. We could not investigate the interaction between choice of analgesia and anesthesia because the two were unmatched. There was no significant interaction between analgesia selection, condition, and the SOZ.

### Classification of the SOZ using intraoperative and sleep HFO rates

**Figure 3:**
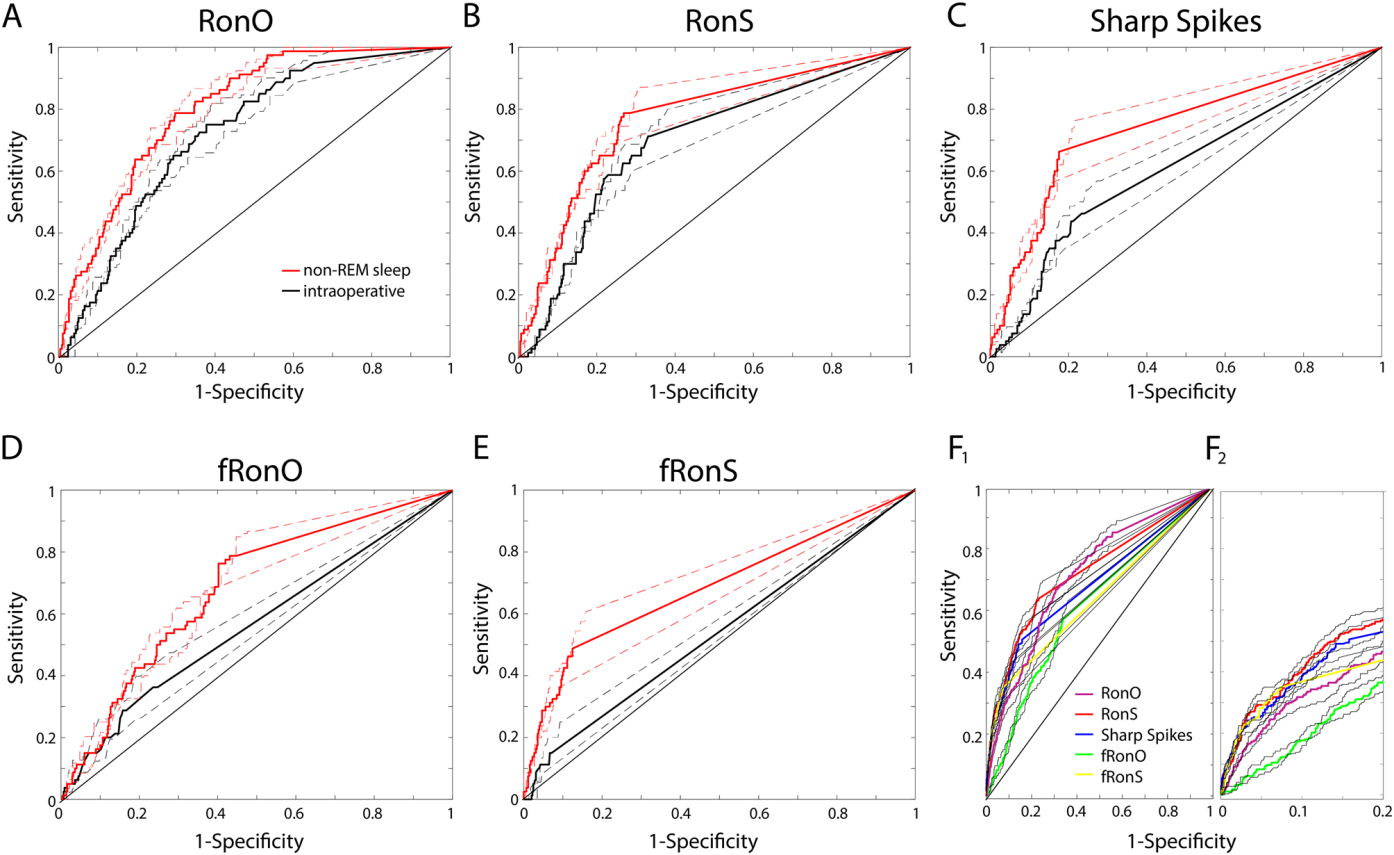
Comparison of receiver-operating characteristic (ROC) curves for different HFO subtype and sharp spike rates (**A**–**E**) in classifying the SOZ during intraoperative recordings (n = 530 contacts, black) and the same matched electrode contacts during non-REM sleep recordings (n = 530, red). Dashed lines indicate 95% confidence intervals (CI) for the ROC curves. Only sharp-spikes and fRonS had a significantly larger area under the ROC curve (AUROC) in the sleep recordings (*p* < 0.05). (**F**) ROC curves for the HFO subtype and sharp spike rates using all contacts (n = 2630), including those unmatched, for classifying the SOZ sites during the non-REM sleep recordings (**F1**). At high specificities (**F2**), the partial AUROC of RonO and fRonO were inferior to sharp-spikes and HFOs on spikes (*p* < 0.5). Black lines indicate 95% CI.

Since HFO rates were increased in the SOZ in the intraoperative and non-REM sleep recordings, we next used receiver-operating characteristic (ROC) curve analysis to assess how well the rate of HFOs on each matched electrode contact (n = 530) during intraoperative and non-REM sleep recordings classified the SOZ. Using boot-strap analysis, we found that the area under the ROC (AUROC) curve was larger for RonO and RonS during non-REM sleep recordings than during the intraoperative recordings, and was significantly larger for sharp-spikes, fRonO, and fRonS (*p* < 0.05, n = 1000 surrogates, Fig. 3A–E). RonO had the largest AUROC and was 0.72 [95% confidence interval (CI) 0.66–0.77] for intraoperative recordings, and 0.80 [95% CI 0.75–0.84] for sleep recordings. During the intraoperative and sleep recordings, the AUROC curve was significantly smaller for fRonO and fRonS than RonO (*p* < 0.05), but this may be due to a plateau effect at a limited specificity seen in the curves for fRonO and fRonS.

Since only a subset of the total number of electrodes were recorded in the intraoperative setting and in some cases, were outside the SOZ, we repeated the ROC curve analysis using rates of HFOs recorded from all electrodes during non-REM sleep (n = 2630), including those that were unmatched. We found that at specificities > 80%, RonO and fRonO were inferior to sharp-spikes and HFOs on spikes for classifying the SOZ (Fig. 3F, *p* < 0.05).

### Differences in the spatial distribution of HFOs and sharp-spikes in the intraoperative and non-REM sleep condition

**Figure 4:**
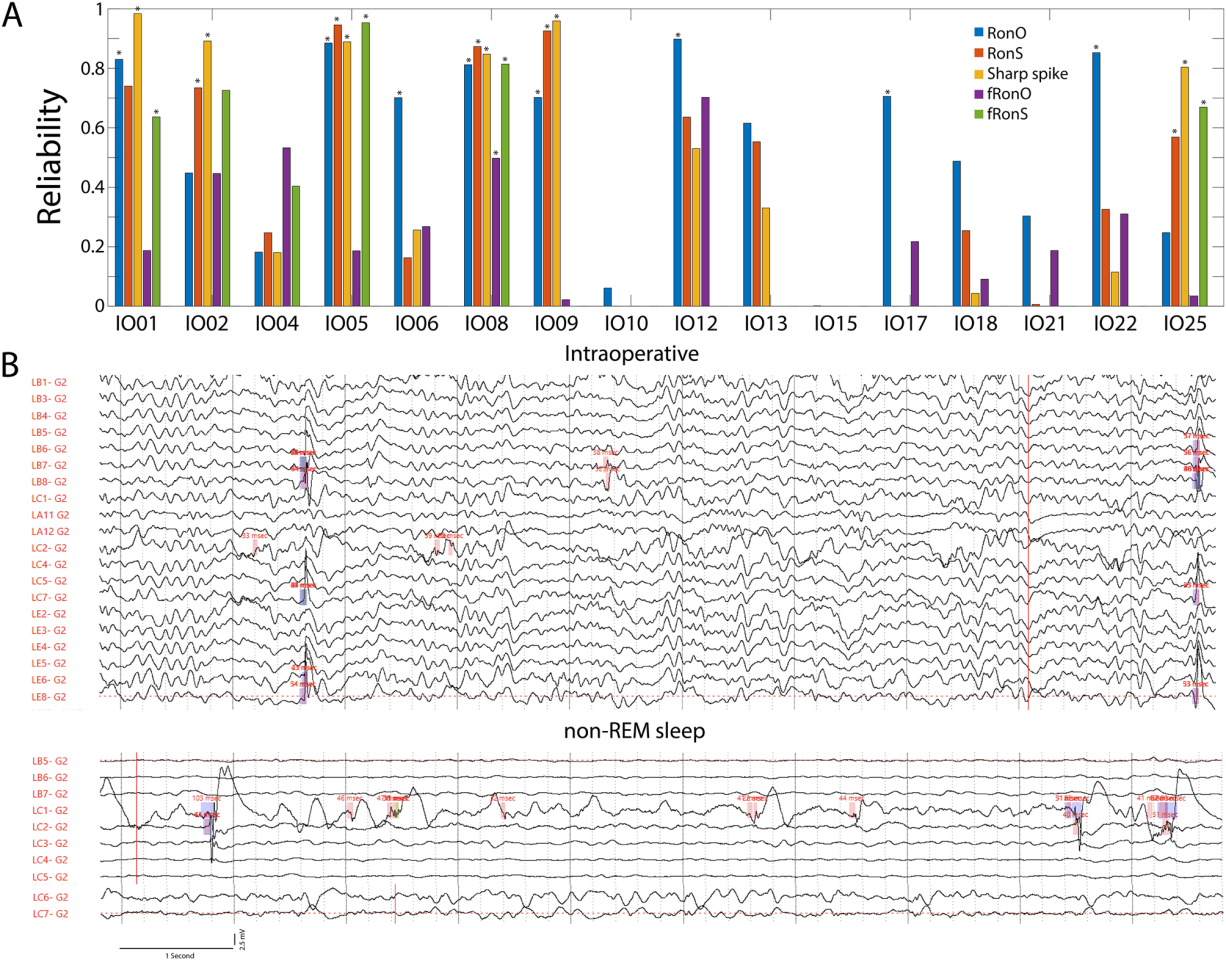
Evaluation of reliability in the spatial distribution of HFOs and sharp-spikes during the intraoperative and non-REM sleep recordings from matched electrode contacts. (**A**) The reliability measure computed from the scalar product for all pairs of HFO vectors between the two conditions for the 16 patients. Asterisk indicates significant reliability (*p* < 0.05) with respect to a random distribution (n = 2000 surrogates). (**B**) Intraoperative recording from patient IO015 shows RonS (pink and blue) in lateral temporal contacts (LB7,8,LC7), but during non-REM sleep recordings RonS, RonOs (pink), and fRonOs (green) appeared on in a mesial temporal contact (LC1), which was part of the SOZ.

To better understand the within-patient differences in HFO generation in the intraoperative and non-REM sleep recordings we calculated the scalar product for all pairs of HFO vectors during the two conditions using the paired contacts. We found that in some patients, HFOs and spikes were very reliable between the two conditions (Fig. 4A, *p* < 0.05, n = 2000 surrogates). However, in other patients, the spatial distribution was less reliable or completely different (Fig. 4B, Figure S6). Overall, fRonO were the least reliable of all the event types across the 16 patients (Fig. 4A).

### Comparison of resection of HFO and sharp-spike positive regions in individual patients with post-operative seizure outcome

To determine if HFOs and sharp-spikes recorded intraoperatively and during sleep define the EZ, we used an established method^6,7,15^ to define the contacts that exhibited the highest rate of events in each patient as the putative EZ. In eight patients, post-resection imaging was available, and we asked if the putative EZ was completely within the resection cavity. Four of the patients were not seizure free after surgery (Table S1). In two of the eight patients the resected region was not sampled by the intraoperative recording. In the intraoperative recordings, we found that the EZ defined by fRonO and fRonS trended the most sensitive for predicting failure. However, specificity and PPV were low (Table 2). Interestingly, these same events recorded from the same matched contacts during sleep performed slightly worse, but this difference was not statistically significant (Table 2). In the sleep recordings from all contacts, including those unmatched, fRonO trended the most sensitive and accurate among all the biomarkers, and also trended better than almost all of the biomarkers in the intraoperative recordings which had a limited montage (Table 2). However, even in the sleep recordings from all the electrodes, two seizure free patients had electrodes with fRonO that were outside the margins of resection.

**Table 2:** Sensitivity, specificity, positive predictive value (PPV), negative predictive value (NPV, and accuracy of remaining HFO and sharp-spike (SSpike) positive regions (see “Methods” section) for predicting surgical failure (Engel Class ≥ 2) in the intraoperative and electrode matched non-REM sleep recordings (n = 6), and the sleep recordings from all contacts (all, n = 8).

	Sensitivity	Specificity	PPV	NPV	Accuracy
RonO intraop	0.67 [0.11–0.88]	0.67 [0.22–0.96]	0.67 [0.22–0.96]	0.67 [0.36–1.0]	0.67 [0.22–0.96]
RonO sleep	0.67 [0.11–0.88]	0.67 [0.22–0.96]	0.67 [0.22–0.96]	0.67 [0.36–1.0]	0.67 [0.22–0.96]
RonO all	1.0 [0.63–1.0]	0.0 [0.0–0.36]	0.50 [0.03–0.65]	0.0 [0.0–0.0]	0.50 [0.09–0.75]
RonS intraop	0.67 [0.12–0.89]	1.0 [0.54–1.0]	1.0 [0.54–1.0]	0.75 [0.23–0.96]	0.83 [0.36–0.99]
RonS sleep	0.33 [0.0–0.64]	0.67 [0.23–0.96]	0.50 [0.12–0.88]	0.50 [0.04–0.78]	0.50 [0.12–0.88]
RonS all	0.75 [0.35–0.97]	0.25 [0.0–0.52]	0.50 [0.16–0.84]	0.50 [0.09–0.76]	0.50 [0.16–0.84]
SSpike intraop	0.67 [0.36–1.0]	0.67 [0.36–1.0]	0.67 [0.11–0.88]	0.67 [0.36–1.0]	0.67 [0.0–0.64]
SSpike sleep	0.33 [0.22–0.96]	0.67 [0.23–0.96]	0.50 [0.12–0.88]	0.50 [0.04–0.77]	0.50 [0.12–0.88]
SSpike all	0.75 [0.35–0.97]	0.25 [0.0–0.52]	0.50 [0.16–0.84]	0.50 [0.09–0.76]	0.50 [0.16–0.84]
fRonO intraop	1.0 [0.54–1.0]	0.33 [0.04–0.77]	0.60 [0.54–1.0]	1.0 [0.54–1.0]	0.67 [0.22–0.96]
fRonO sleep	0.67 [0.54–1.0]	0.33 [0.04–0.78]	0.50 [0.0–0.64]	0.50 [0.0–0.64]	0.5 [0.12–0.88]
fRonO all	1.0 [0.63–1.0]	0.50 [0.16–0.84]	0.67 [0.35–0.97]	1.0 [0.63–1.0]	0.75 [0.63–1.0]
fRonS intraop	1.0 [0.54–1.0]	0.33 [0.0–0.64]	0.60 [0.04–0.77]	1.0 [0.54–1.0]	0.67 [0.12–0.88]
fRonS sleep	0.67 [0.22–0.96]	0.33 [0.12–0.88]	0.50 [0.22–0.96]	0.50 [0.04–0.77]	0.50 [0.0–0.64]
fRonS all	0.75 [0.25–0.92]	0.25 [0.09–0.76]	0.50 [0.35–0.97]	0.50 [0.09–0.76]	0.50 [0.09–0.76]

Brackets indicate 95% confidence intervals.

## Discussion

In summary, intraoperative recordings under anesthesia altered HFO morphology, rate, and in some patients, the spatial distribution compared to HFO from the same electrode contacts recorded during non-REM sleep. Overall, HFOs and sharp-spikes delineated the SOZ more accurately in the non-REM sleep than intraoperative recordings (Table 3). The neuroanatomic location of the SOZ did not significantly influence this effect. When the EZ was defined using all contacts during non-REM sleep, fRonO trended the best predicting post-operative seizure outcome, but the specificity and PPV were relatively low (Table 3). Thus, intraoperative SEEG recording is not a substitute for prolonged non-REM sleep recordings for identifying epileptogenic regions using inter-ictal biomarkers.

**Table 3:** Summary of findings. Minus sign indicates negative change, plus sign indicates positive change, minus and equals sign indicates negative trend, but no significant change.

Bio-marker	Effect of anesthesia on HFO power	Effect of anesthesia on HFO spectral content	Effect of anesthesia (sevoflurane 2%) on event rate	Overall accuracy for SOZ (both conditions)	Sensitivity for SOZ at high specificities (sleep)	Effect of anesthesia on SOZ accuracy	EZ accuracy, (non-REM sleep)
RonO	−	−	−	***	**	−/=	*
RonS	−	−	+	**	***	−/=	*
Sharp−spike	n/a	n/a	+	*	***	−	*
fRonO	−/=	++	−	*	*	−	**
fRonS	−	++	−	*	***	−	*

Single asterisk indicates relatively low value, double asterisk medium value, and triple asterisk relatively high value.

HFOs exhibited decreased power under anesthesia, but the difference did not meet significance for fRonO. Also, ripples exhibited a slight but significant decrease in frequency, while fast ripples a large increase in frequency under anesthesia. Fast ripples are thought to be generated by clusters of pathologically interconnected neurons (PIN clusters)^40^ or out of phase firing of pyramidal neurons^41^. Ripples are generated by synchronized inhibitory post-synaptic potentials^42^. Propofol and isoflurane are both GABA_A_ receptor agonists, but isoflurane also inhibits NMDA receptors^43^. Our results suggest that, during HFO generation, propofol and isoflurane decrease the number of recruited synchronized neurons. In the case of fast ripples, this theory is supported by the notion that event spectral frequency is inversely proportional to the size of the network^44^. More work is required to determine whether these changes alter the specificity of the biomarkers for epileptogenic regions. One possibility is, that in the intraoperative recordings, fast ripples may behave more like ripples generated during non-REM sleep because anesthesia increases the spectral frequency.

HFOs except for RonS exhibited lower rates under anesthesia. Notably, RonS and sharp-spike rates were elevated in patients administered 2% isoflurane. Some studies have shown that higher concentrations of isoflurane increase the rate of spikes^45^, but in our study the increase may have been artifactual due to sharply contoured oscillations that would not have necessarily been marked as spikes on visual review. We did not redact these detections because of the ambiguity of the events, and the coinciding ripple events. Neuroanatomical differences may also have contributed, since RonS and sharp-spike rates were elevated during the intraoperative recording, relative to the sleep recording, in the frontal lobe and to a lesser degree the parietal lobe.

In comparing the anesthetics given in this study, propofol resulted in the largest decrease in HFO and spike rates in the absence of a burst-suppression pattern. A decrease in HFO rates with the use of propofol has been reported previously, and it is recommended to wean the propofol off before recording HFOs^24^. Opioid analgesia also decreased the rate of some HFO types and sharp-spikes. Typically, in electrocorticography recordings, these agents are given as boluses to increase spike rates^46^. In this study these agents were given as a constant infusion which has been shown in other studies to not increase spike rates^47^.

With respect to delineating the SOZ, HFO and sharp-spikes rates were relatively higher in the SOZ during non-REM sleep than during intraoperative recordings. Very few studies have compared the accuracy of intraoperative-recorded spikes and HFOs to localize the SOZ^19,22–24^. In one study^23^, however, HFOs recorded under propofol could define the SOZ, but accuracy between intra- and extra-operative recordings was not performed. Anesthesia could disrupt the normal expression of HFOs and spikes by epileptogenic tissue, while in contrast non-REM sleep facilitates the generation of spikes and HFOs in the SOZ and the EZ^29,48^. Frontal and parietal lobe seizures occur more frequently during non-REM sleep^49,50^, and pathological ripples there preferentially occur during the transition between the up-down state^51–53^. In other regions, such as the hippocampus^10^, the facilitation of pathological HFOs and spikes by the up-down state is less pronounced. To examine potential causes of the observed decrease in accuracy of HFOs for epileptogenic regions in the intraoperative condition, future experiments could examine changes to HFO phase amplitude coupling to slower oscillations under anesthesia.

Among the 8 patients with post-operative MRI, fRonO had the highest accuracy for localizing the EZ, which is consistent with many past studies relating resection of HFOs with outcome^4,6,7,15,32,54,55^. By contrast, fRonO rates had the lowest accuracy of all the HFO types for identifying the SOZ in both intraoperative and sleep recordings. This inconsistency highlights the differences between the SOZ and the EZ, and also the dangers of using the SOZ as a metric by which to evaluate the utility of HFOs for surgical planning^8^. Also, HFOs on spikes and sharp-spike rates were more sensitive for the SOZ at high specificities, but notably these events trended less accurate for delineating that EZ. The study was underpowered to detect significant differences between HFOs on oscillations and HFOs on spikes for delineating the EZ. Several studies have suggested that HFOs on spikes are more accurate^3,54,56^, and are tightly correlated with increases in single unit firing rate^57^. Results are consistent with the hypothesis pathological HFOs, and specifically fRonO, are a electrophysiological biomarker for localizing the EZ in epilepsy surgery.

This study was also underpowered to detect significant differences between the intraoperative and sleep recording of HFO to localize the EZ. The intraoperative recordings trended more accurate than the matched contact sleep recordings, this result may be spurious since the SOZ was often only partially sampled by the limited montage of the intraoperative electrode contacts. The accuracy of fRonO in the sleep recordings using all the contacts trended better than the intraoperative recordings. Thus, fRonOs in sleep recordings, with adequate spatial sampling, may be more accurate than intraoperative recordings for defining the EZ. Another indication that supports this conclusion is that fRonO were the least spatially reliable biomarker between the two recording conditions.

In several patients during intraoperative recordings, high rates of, HFOs on spikes and sharp-spikes were found outside the SOZ. One possible explanation is that these spikes were injury potentials from electrode implantation that have been previously reported in studies of intraoperative depth electrode recordings^58^. However, in the hippocampus, these injury potentials were found to last only 1 min after implantation^59^. In the majority of patients, high rates of ectopic spikes were not present. This is consistent with a prior report that found that spike rates in intraoperative recordings from hippocampal depth electrodes correlated with hippocampal sclerosis^2^. Besides the possibility of injury potentials, another potential cause of the poor spatial reliability of HFOs and sharp-spikes was that the sleep recordings were often longer in duration than the intra-operative recordings. Poor temporal sampling should influence low-rate events such as fRonO and fRonS most, which is consistent with our results.

Results from this study were drawn from relatively few patients. For the GLMM, ANOVA, and ROC analysis we increased statistical power by making contact-matched comparisons. We could not use this approach to analyze the accuracy of HFOs and sharp-spikes for the EZ. Also, less than half of the patients in this study had resections followed by post-operative MRI. A larger study is required to prove that the accuracy of HFOs and sharp-spikes is inferior in the intraoperative condition, and would also help to resolve the effects of specific types and doses of anesthesia on delineating the SOZ and the EZ.

As noted previously only a subset of the total number of electrodes were recorded in the operating room and in some cases, were outside the SOZ and EZ. This could be a reason why fRonOs recorded from all electrodes during sleep more accurately defined the EZ than the fRonOs recorded from the subset of electrodes in the operating room. Future experiments may require recording from all SEEG electrodes, but this should be carefully planned since this would prolong the patient’s exposure to anesthesia while connecting all the electrodes. Alternatively, anesthesia could be weaned while the electrodes are being connected which may also improve delineation of the SOZ and the EZ.

In summary, our results suggest that localization of the SOZ and likely the EZ is more accurate using HFOs recorded during sleep than during HFOs recorded in the operating room (Table 3). An appropriately powered, likely multi-centered, study is needed to verify these results. Anesthesia affects the morphology and rate of HFOs. The utility of intraoperative SEEG recordings could be improved by increasing recording duration, tapering patients off anesthesia, and perhaps utilizing opioid boluses. Prolonged SEEG recordings remain essential because they permit electroclinical correlation of the SOZ. However, for patients who cannot tolerate long-term monitoring or have circumscribed lesions on MRI, the utility of intraoperative SEEG deserves further exploration. A lack of spatial reliability, and the possibility of injury potentials remain a concern.

## Methods

All patients underwent intracranial monitoring with depth electrodes between 2016 and 2018 at Thomas Jefferson University (TJU) for the purpose of localization of the SOZ (Table 1). Intraoperative recordings were obtained from the anesthetized patients 10–15 min following implantation of all SEEG electrodes. In the operating room, 10-min recordings were obtained from 3 to 7 depth electrodes per patient. We connected only a subset of the total number of electrodes in the operating room so as not to prolong the patient’s exposure to anesthesia. Depth electrodes selection was based on the results from the patient’s previous scalp EEG study. One-two days after implantation, for each patient a 10–60 min iEEG recording from all the depth electrodes containing large amplitude, delta-frequency slow waves (i.e., non-REM sleep) was selected for analysis. Only iEEG that was at least 4 h of seizure-free, and was free of low levels of muscle contamination, and other artifacts, was selected.

All recordings were referenced to an iEEG electrode contact positioned in the white matter. Clinical iEEG recordings (0.016–600 Hz) were acquired, at a 2 kHz sampling rate, from the depth electrode contacts using a Nihon–Kohden 256-channel JE-120 long-term monitoring system (Nihon-Kohden America, Foothill Ranch, CA, USA). The study was approved by the TJU institutional review boards (IRB Control #16F.592), and patients gave informed consent prior to participating in this research. All research was in accord with the office for human research protections.

The seizure onset zone was defined by the attending epileptologist for each patient and did not include areas of early propagation. The non-SOZ included all remaining contacts and was often separated from the SOZ by sub-centimeter distances (Table 1). In all the patients the SOZ was not completely sampled by the intraoperative recordings, in 4 patients, the SOZ was not sampled at all by the intraoperative recording contacts.

### HFO detection

HFOs and sharp-spikes were detected in the intraoperative and sleep iEEG using previously published methods^3,34,60^ implemented in Matlab (Mathworks, Natick, MA, USA). In brief, the HFO detector reduced muscle and electrode artifacts in the iEEG recordings using a independent component analysis (ICA)-based algorithm^60^. After applying this ICA-based method, ripples and fast ripples were detected in the referential and bipolar montage iEEG recordings per contact by utilizing a Hilbert detector, in which a 1000th order symmetric finite impulse response (FIR) band-pass filter in the (80–600 Hz) for ripples and (250–600 Hz) for fast ripples was applied, and (ii) a Hilbert transform was applied to calculate the instantaneous amplitude of this time series according to the analytic signal z(t)

1 $$ {\text{z}}\left( {\text{t}} \right) = {\text{a}}\left( {\text{t}} \right){\text{e}} \wedge {\text{i}}\phi \left( {\text{t}} \right) $$

where a(t) is the instantaneous amplitude and ø(t) is the instantaneous phase of z(t). Following the Hilbert transform, the instantaneous HFO amplitude function [a(t)] was smoothed using moving window averaging, the smoothed instantaneous HFO amplitude function was normalized using the mean and standard deviation of the time series, and a custom statistical threshold defined by the skewness of the normalized time series was used to detect the onset and offset of discrete/potential events.

HFO-like events can arise due to Gibb's phenomenon, i.e., high-pass filtering sharp transients, including epileptiform spikes^33^. To distinguish authentic HFOs from authentic HFOs on EEG spikes or spurious HFO due to filter ringing, we used a custom algorithm that performed topographic analysis of time–frequency plots for each HFO^34^. The algorithm also measured the power, spectral content, and duration of each HFO and categorized the HFO as an HFO on oscillation or HFO on spike. Following automatic detection of HFO and sharp-spikes, false detections of clear muscle and mechanical artifact were deleted by visual review in Micromed Brainquick (Venice, Italy).In almost all the patients, less than 3–5% of the detections were deleted^3^.

### Neuroimaging

T1- pre-implant and post-resection MRIs were obtained for each patient. Post-implantation CT scans were then co-registered with the MRIs using Advanced Neuroimaging Tools (ANTs)^61^ with neuroradiologist supervision, using an in-house pipeline (https://github.com/pennmem/neurorad_pipeline). The position of each electrode contact was localized to the Desikan-Killiany atlas^62^. Identification of the electrode contacts in the resection cavity was performed manually in itk-SNAP (http://www.itksnap.org/pmwiki/pmwiki.php).

### Statistics

HFO frequency and power values were fit with generalized linear mixed-effects models in Matlab with contact as the random-effects term, and condition and SOZ as fixed-effects predictors. A comparison of HFO and sharp spike rates, by electrode contact, was carried out in Matlab using the RANOVA function with factors including SOZ, anesthesia or analgesia or neuroanatomic lobe of the electrode contact, and two levels for the recording condition. Receiver operating curves were generated using the perfcurve function in Matlab, and 95% confidence intervals were estimated using 1000 boot-strap replicas. We quantified the reliability of the distribution of HFO rates between the intraoperative recording and extraoperative recording. For each interval pair we computed the normalized scalar product of the spatial distribution of the HFO rates. To test the magnitude of the true scalar product against chance, we permuted (N = 2000) the order of channels for the intra-operative and sleep conditions^15^. For the outcome analysis, HFO and sharp spike positive regions were defined as the channels whose rates exceeded the 95th percentile of the distribution of all contacts during the recording (intra-op, matched sleep contacts, all sleep contacts). We defined as true positive a patient whose event area was not fully located in the margins of the resection and the post-operative seizure outcome was Engel IIa or worse. The positive predictive value was calculated as PPV = TP/(TP + FP), negative predictive value as NPV = TN/(TN + FN), sensitivity = TP/(TP + FN), specificity = TN/(TN + FP), and accuracy = (TP + TN)/N. Estimates of the 95% confidence intervals (CI) used the binomial method.

## Supplementary Information

Supplementary Information.

## Data Availability

The datasets generated during and/or analysed during the current study are available from the corresponding author on reasonable request.
