# Supplementary material for: Accuracy of high-frequency oscillations recorded intraoperatively for classification of epileptogenic regions
Source: Sci Rep. 2021 Nov 1;11:21388. doi: 10.1038/s41598-021-00894-3 (PMC8560764; doi:10.1038/s41598-021-00894-3)
Supplement: Supplementary file 1 — Supplementary Information. [file 41598_2021_894_MOESM1_ESM.docx]

**Supplementary Materials**

Supplemental Figures


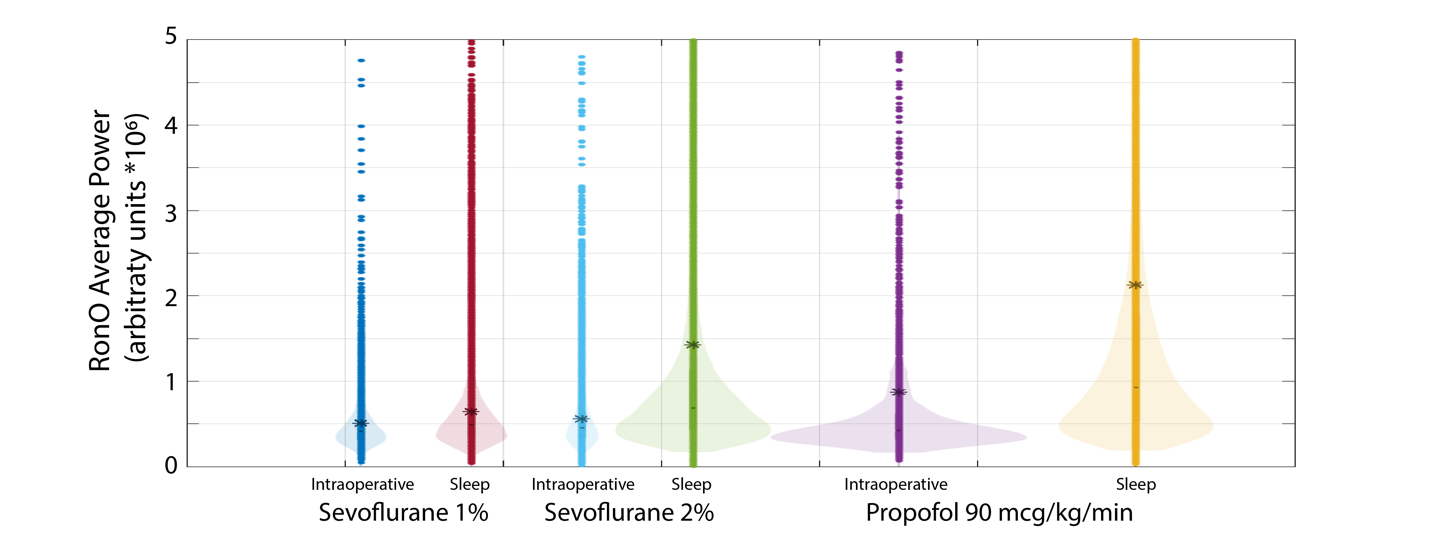


Supplemental Figure 1: Violin plots of the differences in RonO power measured intraoperatively and during non-REM sleep grouped by anesthesia type and dose. The differences between RonO power in the two conditions was relatively larger for sevoflurane 2% and propofol anesthesia compared to sevoflurane 1% anesthesia. However, the difference was relative, and the effect of different anesthetics were not compared across the intraoperative condition. Asterisk indicates mean.


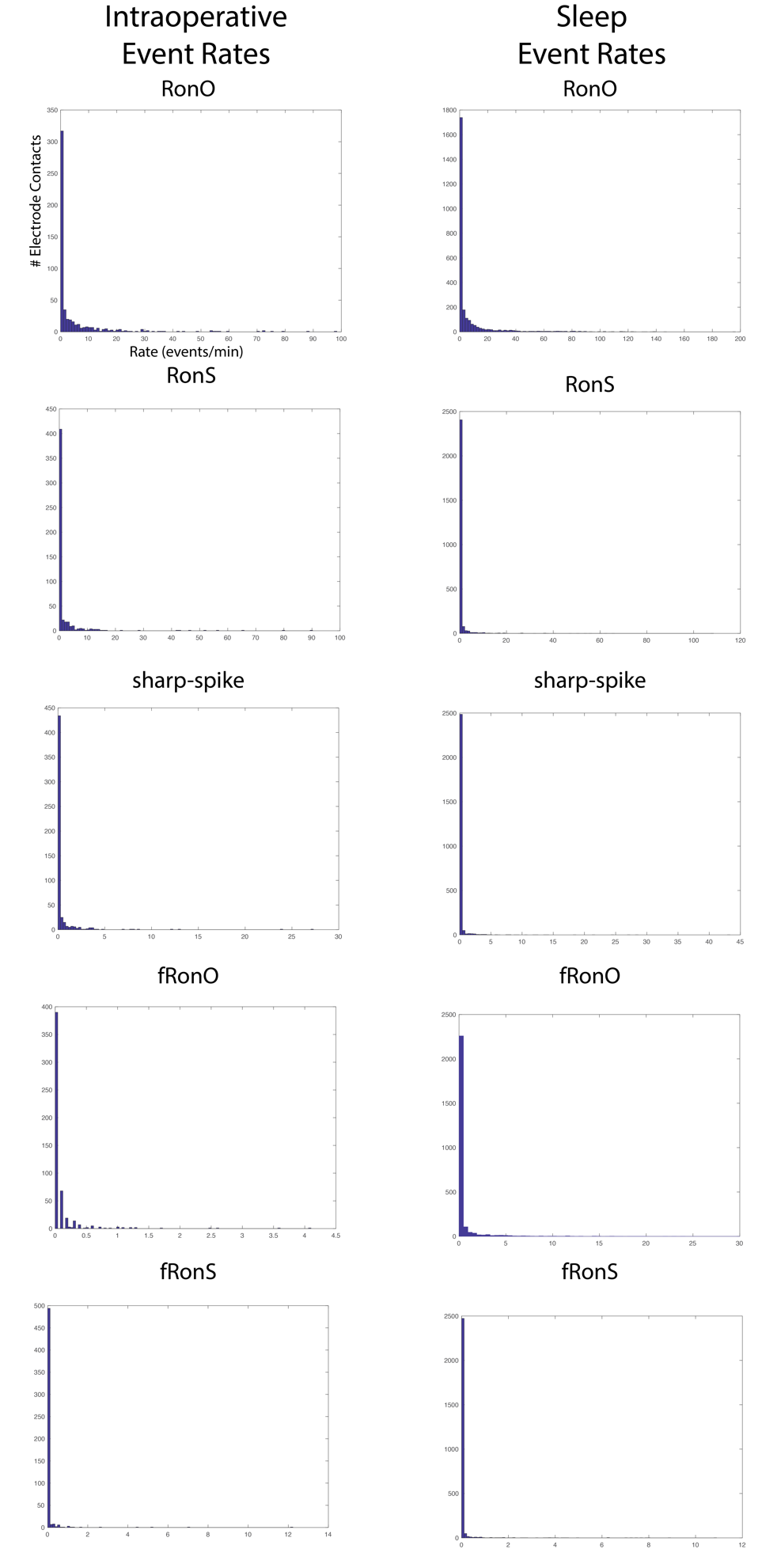
upplemental Figure 2: Histograms of event rates from all electrode contacts used in the intraoperative recordings (left), and all the electrode contacts, including unmatched contacts, during the non-REM sleep recordings (right). Note that most electrode contacts record no events.


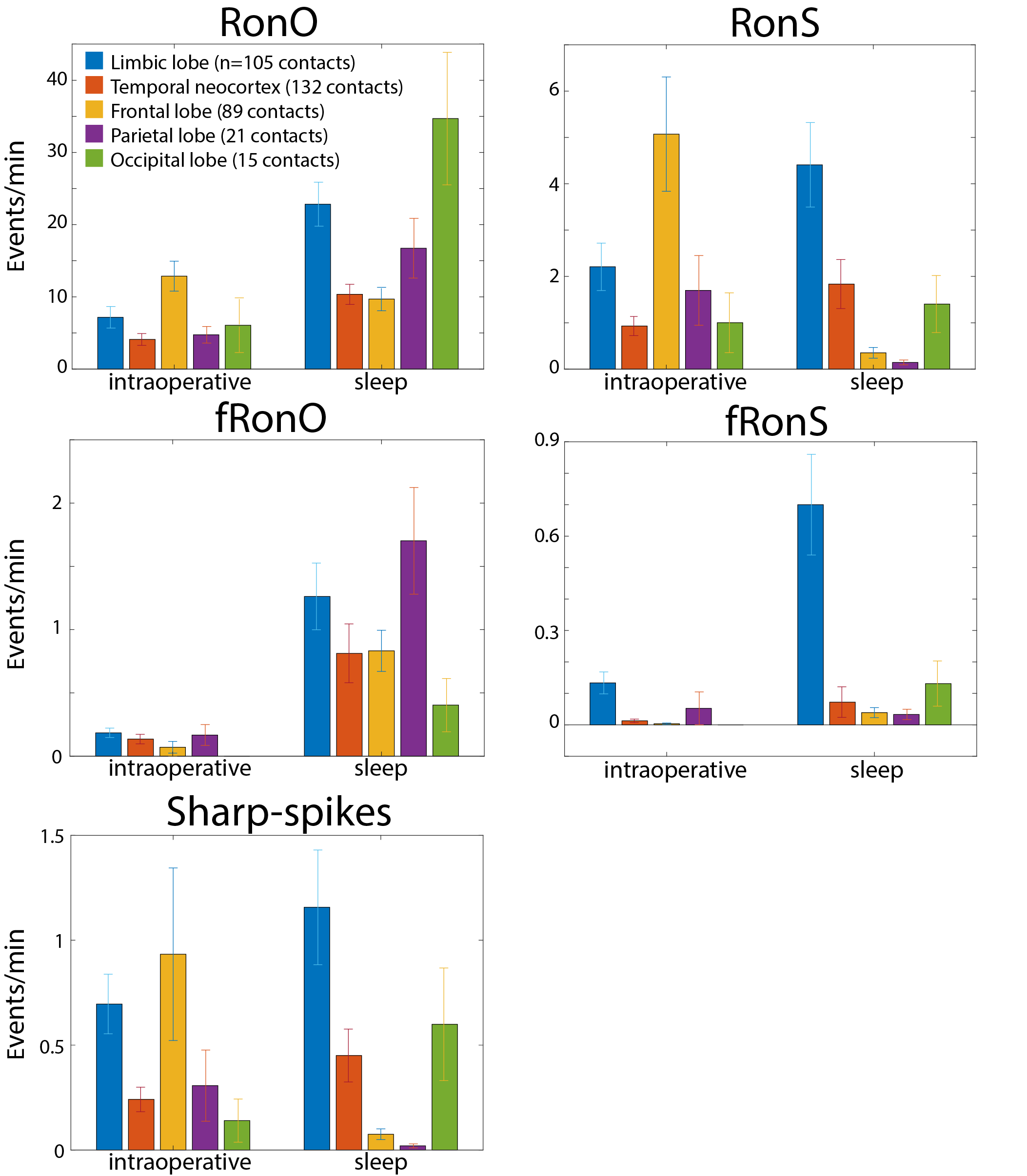


Supplemental Figure 3: Differences in mean HFO and sharp-spike rates between the intraoperative and non-REM sleep condition by the neuroanatomic location of the respective matched recording contacts. Limbic lobe refers to mesial temporal lobe structures and cingulate gyrus. Error bars indicate standard error of the mean (s.e.m). In contrast to other regions, higher ripple and sharp-spike rates, were seen in the frontal lobe, and to a less extent the parietal lobe, in the intraoperative recording relative to the sleep recording.


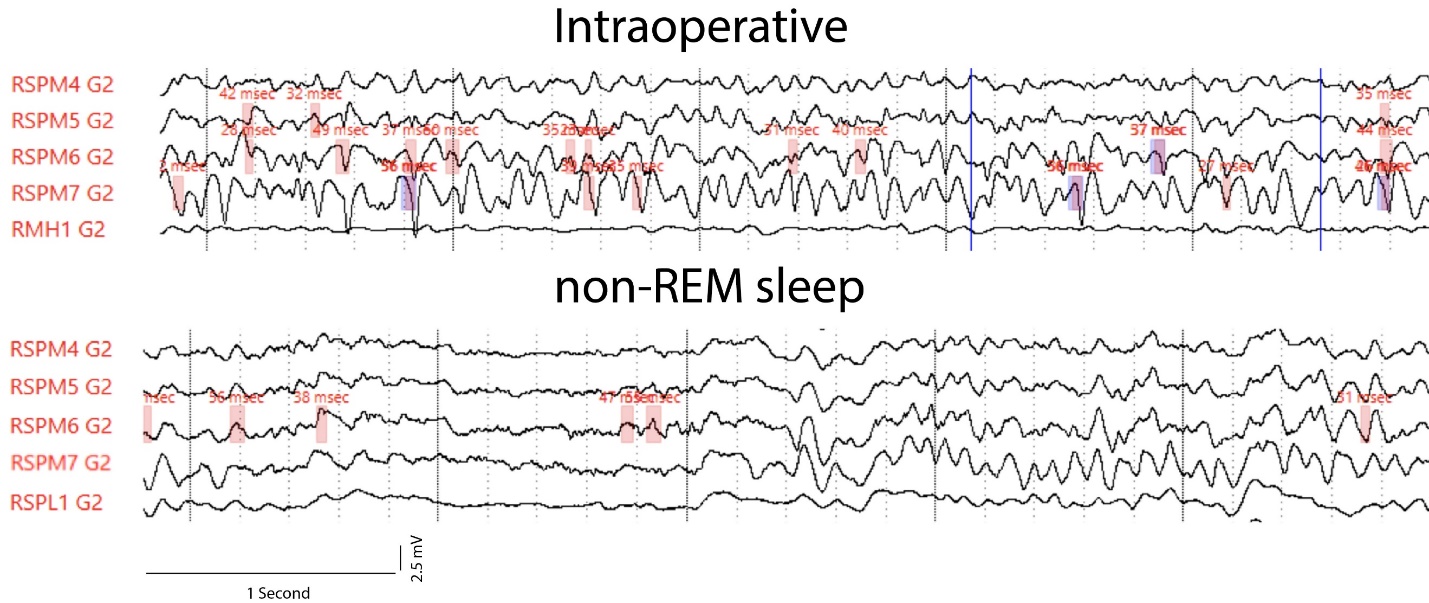


Supplemental Figure 4: Anesthesia administration in patient IO006 resulted in high amplitude sharply contoured theta and alpha oscillations in neocortex that are occasionally recognized as spikes by the automated detector in contact (RSPM7). Ripple oscillations occur during the epochs marked in pink. Ripple on spikes occur during the epochs marked in blue and pink. During extra-operative sleep in this patient, spikes were not recognized in recordings from these electrode contacts.


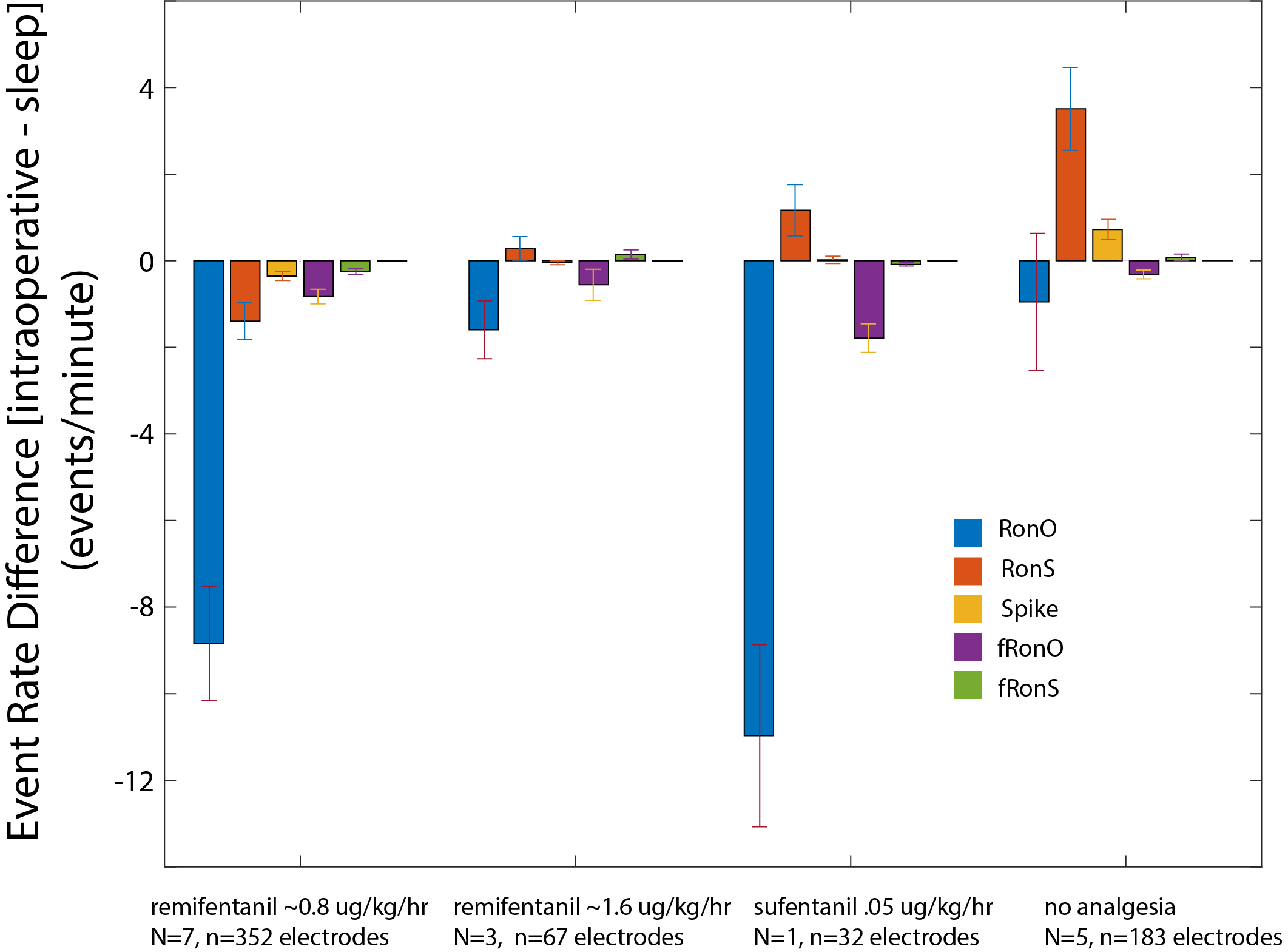


Supplemental Figure 5: Differences in HFO and spike rates in the intraoperative recordings relative to the contact matched sleep recordings by analgesia type and dose. Analgesia type and dose interacted with recording condition (intraoperative vs. non-REM sleep) to influence HFO rates for all HFO subtypes except RonO (p<0.05). Patients with no analgesia did receive fentanyl 50 ug at induction.


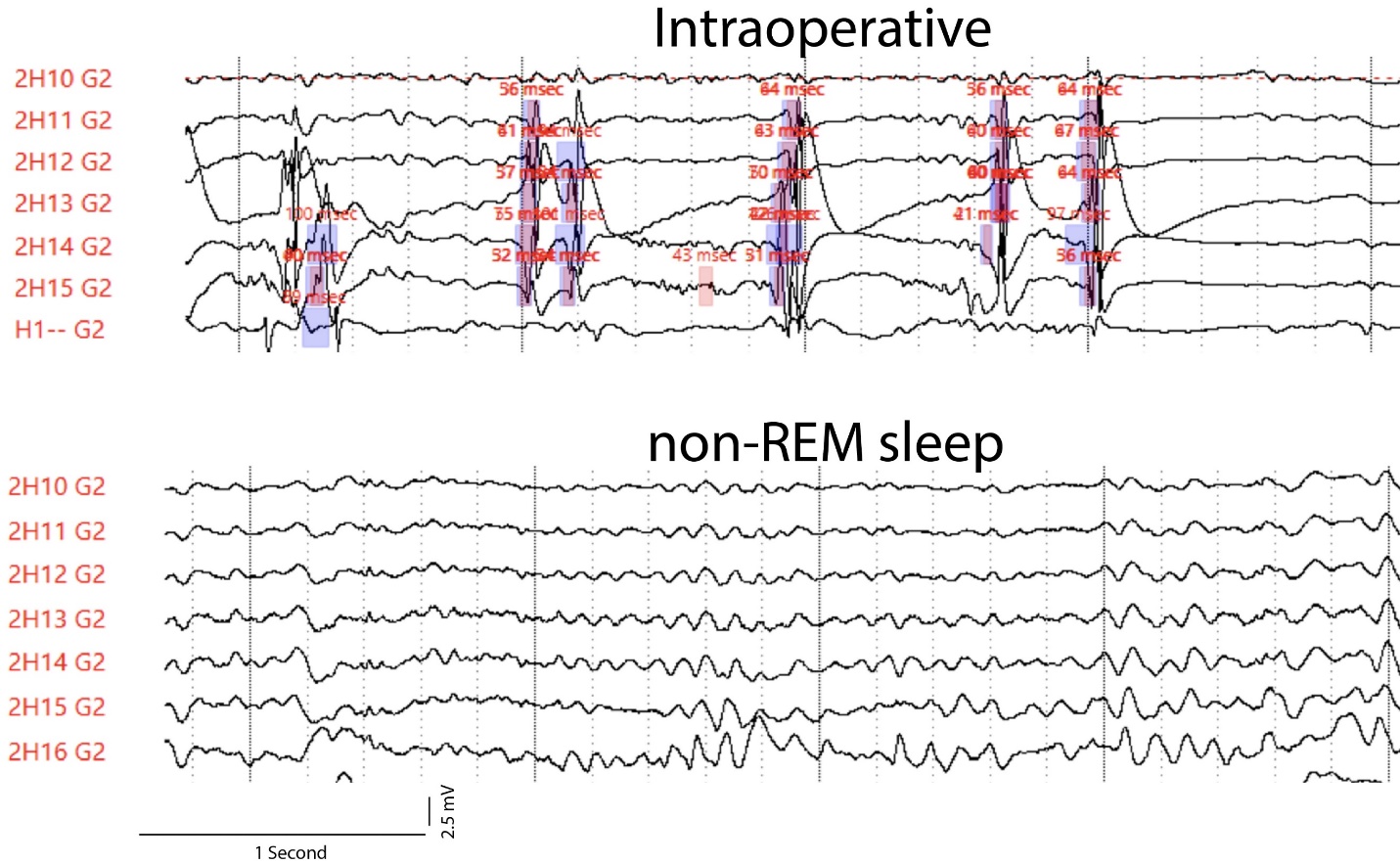


Supplemental Figure 6: Intraoperative recordings from patient IO021 demonstrated periodic 1-2 Hz RonS (red and blue) and spikes (blue) in electrode contacts that were not part of the SOZ and exhibited no pathological activity during non-REM sleep.

Supplemental Tables

Table S1: Patient characteristics: Abbreviations M: male, F: female, L: left, R: right, ipsi: ipsilateral to SOZ, contra: contralateral to SOZ, N/A: not applicable, ATL: anterior temporal lobectomy, MT: mesial temporal, RNS: Responsive neurostimulator. Bolded patient names indicate the patient was included in the analysis of the epileptogenic zone. Listing of contact numbers in the SOZ column refers to the number of intraoperative contacts ipsilateral or contralateral to the SOZ.

| **ID / age / sex** | **Risk Factor** | **MRI** | **PET hypo-metabolic** | **Anesthesia** | **iEEG SOZs**  **# non-SOZ intraop**  **ipsi/contra contacts** | **Surgery** | **Path.** | **Outcome** |
| --- | --- | --- | --- | --- | --- | --- | --- | --- |
| **IO01**  55/F | minor TBI | Normal | L temporal | sevoflurane 2%, remifentanil 0.08 mcg/kg/min | L MT  NSOZ:  19 ipsi,7 contra contacts | modified L ATL | Gliosis | Engel IA@24 months |
| **IO02**  56/M  R | TBI w/ LOC | left hip- pocampal atrophy | R temporal | sevoflurane 2%, remifentanil 0.08 mcg/kg/min | L and R MT, 20 contacts | R ATL | Gliosis | Engel IA@24 months |
| IO04 29/M | none | Normal | L temporal and occipital | sevoflurane 2%, remifentanil 0.08 mcg/kg/min | L lingual gyrus, middle occipital gyrus, 20 ipsi contacts | L occipital lobe resection | cortical dysplasia | Engel IA@ 30 months |
| **IO05** 42/M | febrile seizures | prior hippocampal sparing temporal lobectomy | N/A | sevoflurane,  1.1%,  remifentanil 0.07 mcg/kg/min | R anterior cingulate, MT, uncus,  43 ipsi contacts | R ATL | Gliosis | Engel IVB@40 months |
| IO06 34/M | minor head injury no LOC | Normal | N/A | sevoflurane, 0.9%.  remifentanil  0.05 mcg/kg/min | R cingulate gyrus, SMA, post-central gyrus, precuneus, superior parietal lobule 37 ipsi, 16 contra contacts | RNS R parietal lobe | N/A | Engel IIIA@ 12 months |
| **IO08** 22/F  4161 | other | L temporal lobe atrophy and cortical dysplasia | L temporal | propofol 90 mcg/kg/min, remifentanil 0.10 mcg/kg/min | L temporal lobe lesion  27 ipsi, 21 contra contacts | modified L temporal lobectomy | cortical dysplasia | Engel IA@40 months |
| IO09  20/M | tuberous sclerosis | focal cortical dysplasia, nodules, giant cell tumors | L temporal | propofol 90 mcg/kg/min, remifentanil 0.10 mcg/kg/min | left temporal neocortical, right MT, right precentral gyrus. 34 contacts | None | N/A | N/A |
| IO10 23/M | septo-optic dysplastia | R periventricular pachygyria, deficiency of septum pellucidum | N/A | sevoflorane 1.7%, remifentanil 0.15 ug/kg/min | R SMA, frontal lesion, superior parietal lobule, 19 ipsi contacts | R frontal resection subpial transection | Gliosis | Engel IVB@35 months |
| **IO12**  31/F  4187 | none | 1 cm pineal cyst | R lateral temporal | sevoflourane 2%, remifentanil 0.15 mcg/kg/min | L mesial temporal, 8 ipsi, 20 contra | Modified L temporal lobectomy | Gliosis | Engel IIB@24 months |
| IO13 40/M | None | L parietal lobe resection | L parietal and L occipital | sevoflurane 2.3 %, | L insula, precuneus, middle occipital gyrus, superior parietal lobule, superior occipital gyrus, superior temporal gyrus, middle temporal gyrus, 29 ipsi contacts | left parietal, posterior temporal, occipital resection | Gliosis | Engel IIIA@18 months |
| IO15 37/M | None | L posterior fossa arachnoid cyst, prior right ATL | R temporal | sevoflurane 2.2%, remifentanil 0.15 ug/kg/min | L MT, R cingulate, post. cingulate, mesial frontal, precuneus,  20 left contacts | Right cingulate thermal ablation | N/A | Engel IVB@36 months |
| IO17  29/M | None | Non-specific linear FLAIR signal abnormality in the RT sub-cortical white matter. | R temporal | sevoflurane 2.4%,  fentanyl 50ug | Left mesial temporal, right occipital  32 contacts | Right temporal and occipital resection | Gliosis | Engel IA@36 months |
| **IO18**  28/M | Minor TBI | Normal | Normal | sevoflurane 2.3%,  fentanyl 50ug | Right insula, cuneus, inferior and middle frontal gtrus, 41 ipsi contacts | R. Frontal lobe | Gliosis | Engel IA @ 24 months |
| **IO21**  50/F | None | R frontal subcortical white matter abnormality  Prior R ATL resection | N/A | sevoflurane 2% mac,  fentanyl 50ug | Right Mesial temporal, Right orbitofrontal cortex. 39 ipsilateral | R. Frontal lobe | hippocampal sclerosis, cortical dysplasia | Engel IVB @ 24 months |
| IO22 48/M | None | scattered white matter hyperintensities | bilateral temporal hypometabolism | sevoflurane 1.9%,  sufentanil .05ug/kg/hr | bilateral cingulate gyrus, L middle frontal hyrus, middle frontal gyrus, SMA, pre-central gyrus, post-central gyrus,36 contacts | Anterior corpus callosotomy | N/A | Engel IVB@24 months |
| IO25  47/F | None | tiny gray matter heterotopia adjacent to the left ventricular atrium, hippocampi appear malrotated | Right temporal hypometbaolism. | sevoflurane 2%,  fentanyl 50ug | Bilateral mesial-temporal, 42 contacts | VNS | N/A | Engel IVB@24 months |

Table S2: Results of generalized linear mixed-effects models fitting RonO power in patients receiving different types and doses of anesthesia. The random-effect term was electrode, the fixed effects were recording condition (intraoperative vs. non-REM sleep), and SOZ. Brackets indicate 95% confidence interval. Sleep was correlated with larger increased in RonO power in patients receiving 2% sevoflurane and propofol, compared to 1% sevoflurane.

| **Response Variable** | **Intercept estimate** | **Intercept p-value** | **Condition estimate** | **Condition p-value** | **SOZ estimate** | **SOZ p-value** | **d.f.** |
| --- | --- | --- | --- | --- | --- | --- | --- |
| RonO Power  1% sevoflurane | 13.067  [12.9-13.2] | <1e-4 | 0.233  [.17-.29] | <1e-4 | 0.158  [-.09-0.4] | 0.22 | 44724 |
| RonO Power  2%  Sevoflurane | 12.221  [12.0-12.4] | <1e-4 | 1.21  [1.07-1.35] | <1e-4 | 0.40  [0.17-0.63] | <1e-4 | 87995 |
| RonO Power  Propofol  90mcg/kg/min | 12.538  [12.3-12.8] | <1e-4 | 1.28  [1.14-1.43] | <1e-4 | 0.157  [-.27-.59] | 0.48 | 63751 |

Table S3: Results from the repeated measure ANOVA comparing HFO and sharp-spike rates in matched localized electrode contacts (d.f.=352) between the intraoperative and non-REM sleep conditions (d.f.=1) with the SOZ (d.f.=1) and neuroanatomic lobe (d.f.=4) of the electrode contact as factors. Abbreviations (n.s.: not significant).

|  | Location  F, pValue | SOZ:Location  F, pValue | Location:Condition  F, pValue | SOZ:Location:Condition  F, pValue |
| --- | --- | --- | --- | --- |
| RonO Rates | 5.198,  <1e-4 | 3.564  <1e-2 | 14.093  <1e-10 | 1.206  n.s. |
| RonS Rates | 3.157  <5e-2 | 3.964  <1e-2 | 8.032  <1e-5 | 1.629  n.s. |
| Sharp-spike Rates | 3.883  <1e-2 | 3.877  <1e-2 | 4.271  <1e-2 | 1.205  n.s. |
| fRonO Rates | 2.653  <5e-2 | 2.134  n.s. | 1.685  n.s. | 1.610  n.s. |
| fRonS Rates | 10.207  <1e-7 | 2.011  n.s. | 8.510  <1e-5 | 2.102  n.s. |
